# Correction: Estimating Potential Infection Transmission Routes in Hospital Wards Using Wearable Proximity Sensors

**DOI:** 10.1371/annotation/b20d3cec-62b7-44ec-9150-8a06a9b30a9b

**Published:** 2013-09-20

**Authors:** Philippe Vanhems, Alain Barrat, Ciro Cattuto, Jean-François Pinton, Nagham Khanafer, Corinne Régis, Byeul-a Kim, Brigitte Comte, Nicolas Voirin

Multiple funding organizations and grants were incorrectly listed in the Funding Statement. In addition, multiple funding organizations and grants were incorrectly omitted from the Funding Statement. The Funding Statement should read: "This study was partially supported by the FINOVI foundation, the INSERM IMI Pandemic program and GOJO (Goldie et Jerry, GOJO Industries, Inc.). A.B. is partially supported by the FET project Multiplex 317532 and by ANR project HarMS-flu (ANR-12-MONU-0018). C.C. is partially supported by the FET project Multiplex. The funders had no role in study design, data collection and analysis, decision to publish, or preparation of the manuscript." 

